# Novel Human *FCGR1A* Variants Affect CD64 Functions and Are Risk Factors for Sarcoidosis

**DOI:** 10.3389/fimmu.2022.841099

**Published:** 2022-03-17

**Authors:** Jianming Wu, Yunfang Li, Aaron Rendahl, Maneesh Bhargava

**Affiliations:** ^1^ Department of Veterinary and Biomedical Sciences, College of Veterinary Medicine, University of Minnesota, St. Paul, MN, United States; ^2^ Division of Pulmonary, Allergy, Critical Care & Sleep Medicine, Department of Medicine, School of Medicine, University of Minnesota, Minneapolis, MN, United States

**Keywords:** FCGR1A genetic variants, CD64 expression, phagocytosis, degranulation, sarcoidosis

## Abstract

CD64 (or FcγRIA) is the sole functional high affinity IgG Fc receptor coded by *FCGR1A* gene in humans. The *FCGR1A* genetics has not been comprehensively investigated and effects of human *FCGR1A* variants on immune functions remain unknown. In the current study, we identified three novel *FCGR1A* variants including the single nucleotide variant (SNV) rs1848781 (c.-131) in the proximal *FCGR1A* gene promoter region, the rs587598788 indel variant within the *FCGR1A* intron 5, and the non-synonymous SNV rs1050204 (c.970G>A or FcγRIA-p.D324N) in the *FCGR1A* coding region. Genotype-phenotype analyses revealed that SNV rs1848781 genotypes were significantly associated with CD64 expression levels. Promoter reporter assays show that rs1848781G allele had significantly higher promoter activity than the rs1848781C, confirming that the rs1848781 is a functional *FCGR1A* SNV affecting promoter activity and gene expression. The rs587598788 indel genotypes were also significantly associated with levels of CD64 expression. Moreover, the non-synonymous SNV rs1050204 (FcγRIA-p.D324N) alleles significantly affected CD64-mediated phagocytosis, degranulation, and pro-inflammatory cytokine productions. Genetic analyses revealed that *FCGR1A* genotypes were significantly associated with sarcoidosis susceptibility and severity. Our data suggest that *FCGR1A* genetic variants may affect immune responses and play a role in sarcoidosis.

## Introduction

IgG Fc receptors (FcγRs) play critical roles in regulating immune responses (1, 2). FcγRs mediate a variety of immune functions including phagocytosis (3), antigen presentation (4), degranulation (5), cytokine production (6), and immune complex clearance (7). FcγRs also play important roles in inflammation (8, 9). Human genome has three highly homologous *FCGR1* (CD64) gene family members (*FCGR1A*, *FCGR1B*, and *FCGR1C*) (10, 11) among which *FCGR1A* is the sole functional gene capable of producing a high-affinity FcγR CD64 (12, 13). Due to the nearly identical nucleotide sequences among *FCGR1* gene family members, the genetics of *FCGR1A* has never been systemically investigated and the genetic markers within the human *FCGR1A* are not identified. Accordingly, it remains a mystery whether *FCGR1A* coding for CD64 contains functional genetic variants influencing the pathogenesis of inflammatory diseases.

Sarcoidosis is a multisystem granulomatous disorder that often leads to poor lung functions and high morbidity (14). Environmental factors are initiators for the disease but the definitive trigger has not been identified for sarcoidosis (15, 16). On the other hand, twin studies, disease clustering in families, and racial differences in incidence rates point to the effect of genetic risk factors on the development of sarcoidosis (17–22). Both MHC genes and non-MHC genes were found to associate with sarcoidosis (21–26). The powerful genome-wide association studies (GWAS) revealed several genes involved in sarcoidosis (25, 26). Nevertheless, GWAS lacks the sensitivity to precisely determine causal variants and explains only a small proportion of the expected heritability (27). Our previous study demonstrated that copy number variations (CNVs) of low affinity FcγRs are major risk factors for sarcoidosis (28), suggesting that the high affinity IgG receptor coded by *FCGRIA* gene may also play a role in sarcoidosis.

In the current study, we have identified three novel variants within the *FCGR1A* gene. *FCGR1A* variants significantly affected CD64 expression and functions. Moreover, *FCGR1A* variants were associated with sarcoidosis susceptibility and disease severity. Our data suggest that *FCGR1A* genetic variants could affect immune responses and development of sarcoidosis.

## Materials and Methods

### Study Subjects

Healthy American blood donors (n = 392) were recruited at the Memorial Blood Center (737 Pelham Boulevard, St. Paul, Minnesota 55114) as previously described (29). The age of healthy blood donors ranged from 19 to 84 years-old with the mean age of 64 ± 13.5 years and >98% of donors in the study were self-declared Caucasians living in the State of Minnesota. Sarcoidosis patients (n = 59) for CD64 expression phenotype analysis were recruited at the University of Minnesota Medical Center Interstitial Lung Disease Clinic. The ages of sarcoidosis patients ranged from 28.7 to 79.2 years-old with the mean age of 55.4 ± 13.7 years. In addition, human samples and data from the ACCESS (A Case Control Etiologic Study of Sarcoidosis) cohort were used in the genetic association analysis. The DNA specimens and data of the ACCESS sarcoidosis patients (670 cases) and healthy controls (669 controls) were provided by the NHLBI Biologic Specimen and Data Repository as previously described (28). The human study was approved by the Institutional Review Board for Human Use at the University of Minnesota (IRB Protocol #1301M26461).

### Evaluation of CD64 Expressions on Monocytes

The expressions of CD64 on monocytes were determined with flow cytometry analysis with fluorescent-labeled monoclonal antibodies from BioLegend (San Diego, CA). Leukocytes stained with either FITC-conjugated anti-CD64 mAb (10.1, mIgG1) plus APC-conjugated anti-CD14 or mIgG1-FITC isotype control plus APC-conjugated anti-CD14 were analyzed on a FACS Canto flow cytometer (BD Biosciences). The FlowJo software (Tree Star Inc.) was used to evaluate flow cytometry data. Characteristic light-scatter properties were used to identify neutrophils and CD14^+^ monocytes cells were gated in flow cytometry for the geometric mean intensity of CD64 expression.

### Nucleic Acid Isolation

Human genomic DNA was isolated from EDTA anti-coagulated peripheral blood using the Wizard Genomic DNA Purification kit (Promega, Madison, WI) by following the vendor’s instruction.

### *FCGR1A* Sequence Analysis

**Figure 1:**
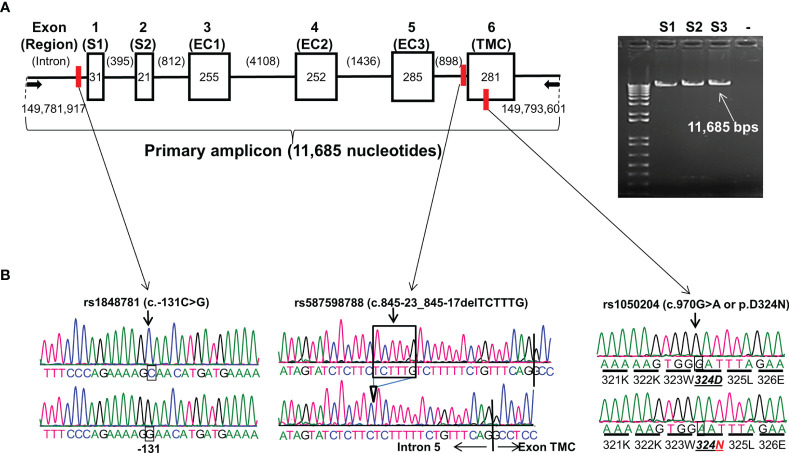
*FCGR1A* gene structure and variants. **(A)** *FCGR1A* gene (NC_000001.11 Chromosome 1 Reference GRCh38.p13 Primary Assembly 149,782,694..149,791,675) at Chromosome 1q21.2 contains 6 exons encoding a protein peptide of 374 amino acids. Arrows indicate the positions of upper primer (starting at nucleotide position 149,781,917 on Chromosome 1 GRCh38.p13 Primary Assembly) and lower primer (ending at nucleotide position 149,793,601 on Chromosome 1 GRCh38.p13 Primary Assembly) in *FCGR1A* PCR amplification. Six boxes (▯) represent six exons with their respective nucleotide coding sequence lengths in bps. Five introns (—) with their nucleotide sizes in bps are shown schematically. The primary PCR amplicon of 11,685 nucleotides covered 1,125-nucleotide coding sequence, 51-nucleotide 5′-UTR, and 159-nucleotide 3′-UTR of *FCGR1A*. The red vertical bars indicate the positions of the three *FCGR1A* variants. S1 and S2 are two exon encoding the FcγRIA signal peptide. FcγRIA extracellular domain 1 (EC1), 2 (EC2), and 3 (EC3) are coded by the exon 3, 4, and 5, respectfully. The exon 6 codes for the FcγRIA transmembrane segment and cytoplasmic domain (TMC). **(B)** Sequence tracers of three *FCGR1A* variants. Arrows indicate locations of SNV rs1848781 (c.-131C>G), rs587598788 (c.845-23_845-17delTCTTTG) indel variant, and SNV rs1050204 (c.970G>A or p.D324N) on sequence tracers.

LaserGene DNAstar MegAlign software (Madison, WI) was used to identify the *FCGR1A* gene-specific sequence regions within the *FCGR1A* promoter and 3’-UTR through alignment of nucleotide sequences of *FCGR1A* (RefSeq AL591493), *FCGR1B* (RefSeq AL357493.8), and *FCGR1C* (RefSeq AL109948.9). The long-template PCR with the sense primer (5’-TTA GCT CTC TTT AGC TCT CTT TTT TTA GCT CTC AT -3’) and antisense primer (5’- CCT CGG TAG GTC CCA GGG AGA AGA AAG ATT C -3’) was carried out to produce the *FCGR1A* gene-specific DNA fragment (11,685 bps) containing the proximal promoter region and all six exons of *FCGR1A* (**Figure 1**). PCR was performed using 200 ng genomic DNA, 200 nM of each primer, 200 μM of dNTPs, 1× PrimeStar GXL buffer, and 2 U of PrimeStar GXL DNA polymerase (TaKaRa cat# R050), starting with 98°C for 1 min; 30 cycles of denaturing at 98°C for 10 s, annealing and extension at 68°C for 5 min; with a final extension at 68°C for 7 min on an ABI Veriti 96-well Thermal Cycler. PCR products were treated with ExoSAP-IT (Affymetrix, Santa Clara, CA) before being sequenced on an ABI 3730xl DNA Analyzer with BigDye Terminator Cycle Sequencing Kit. Five sequencing primers were used for sequence analyses: 1) 5’-CAG CCA CAG CCT GTA CCC TT-3’ for the proximal promoter region and exon 1 coding for the signal peptide, 2) 5’-CAC AAA AGC CTC ACC AGT TGC-3’ for the extracellular domain 1 (EC1), 3) 5’-CTT GGG CCT CCT TGT ACC TCC-3’ for the extracellular domain 2 (EC2), 4) 5’-GGT AAA GGG CAT GTC TTT TGT GA-3’ for the extracellular domain 3 (EC3), and 5’-ATG TTT GTA CGC AGT GCT CA-3’ for the transmembrane segment and intracellular domain (TMC). Nucleotide sequence tracers were aligned and compared with the *FCGR1A* genomic sequence (RefSeq NG_007578.1) using the DNASTAR software (DNAStar, Madison, WI) for the identification of *FCGR1A* variants. All variations are described according to current mutation nomenclature guidelines (30), assigning the A of the first ATG translational initiation codon as nucleotide +1 in the *FCGR1A* mRNA coding region (RefSeq NM_000566.3). Genomic DNA samples of 102 human subjects from the cohort of 392 healthy blood donors were used for *FCGR1A* sequence analysis.

### *FCGR1A* Promoter Reporter Constructs

The *FCGR1A* promoter reporter constructs were generated by cloning a *Kpn* I/*Bgl* II-flanked *FCGR1A* promoter DNA (819 bps) fragment into pGL4.23[luc2/minP] vector (Cat# E841A, Promega, Madison, WI). The *Kpn* I/*Bgl* II-flanked DNA products were generated by PCR amplification of the genomic DNA from a SNV rs1848781 (c.-131C>G) heterozygous donor using upper primer 5’-CGG GGT ACC TCT TTA GCT CTC TTT TTT TAG CTC TCA-3’ (underlined and bold nucleotides are *Kpn* I cutting site, the primer anneals at position from -794 to -831) and lower primer 5’-CGC AGA TCT GTT GTC TCC AAG CTG GTG GG-3’ (underlined and bold nucleotides are *Bgl* II cutting site, the primer anneals at position from -20 to -1). The nucleotide sequences of the cloned constructs were confirmed by direct sequencing from both directions on an ABI 377 Sequencer with ABI BigDye Terminator Cycle Sequencing Kit.

### Transient Transfection and Luciferase Assays

Human monocytic cell line U-937 (ATCC# CRL-1593.2) was obtained from ATCC (Manassas, VA). The cells were maintained in the RPMI-1640 medium supplemented with 10% fetal calf serum and L-glutamine (2 mM). The transient transfections were carried out in a 12-well tissue culture plate (Corning). The cells (2 × 10^5^ cells per well) in 2 ml culture medium were transiently transfected with 3 μl of TransIT-2020 reagent (Mirus Bio LLC, Madicon, WI), 1 μg reporter construct plasmid DNA, and 0.025 μg pRL-SV40 plasmid DNA (Promega, Madison, WI) by following vendor’s instruction. The transfected cells were cultured for 20 hours in the presence of IFN-γ (100 U/ml) or absence of IFN-γ before being centrifuged and washed twice with PBS (pH 7.4). The cells were lysed in the wells with the addition of 200 μl of 1× lysis buffer for the Luciferase Assay Systems (Promega, Madison, WI). The cell supernatants were used for luciferase reporter assays by following vendor’s instruction. Relative luciferase light units, standardized to Renilla luciferase activities in dual luciferase reporter assays, are reported as the mean of triplicate samples.

### Generation of the FcγRIA Expression Constructs

The human FcγRIA (CD64) expression constructs were generated by cloning *EcoR I*/*Bam* HI-flanked RT-PCR products with the entire FcγRIA coding region into the cDNA Cloning and Expression Lentivector pCDH-MSCV-MCS-IRES-copGFP (System Biosciences, Mountain View, CA). The *Kpn* I/*Bam* HI-flanked FcγRIA cDNA was amplified from human mixed mononuclear cell cDNA synthesized with the SuperScript^TM^ Preamplification System (Gibco BRL) with the sense primer (5’-CGC GAA TTC GGA GAC AAC ATG TGG TTC TTG ACA A-3’) (underlined and bold nucleotides are *EcoR I* cutting site) and anti-sense primer (5’-CCG GGA TCC CTA CGT GGG CCC CTG GGG CTC CTT -3) (the underlined and bold nucleotides is *Bam* HI restriction cutting site). The PCR reaction was performed with 2 μl of cDNA, 200 nM of each primer, 200 μM of dNTPs, 2.0 mM of MgSO_4_, and 1 U of Platinum *Taq* DNA polymerase High Fidelity (Invitrogen) in a 25 μl reaction volume. The ABI Veriti 96-well Thermal Cycler was used for the PCR reaction starting with 94°C for 3 min, 35 cycles of denaturing at 94°C for 30 s, annealing at 56°C for 45 s, extension at 68°C for 1 min and 30 s with a final extension at 72°C for 7 min. The QuikChange Site-Directed Mutagenesis kit (Stratagene, La Jolla, CA) and two mutagenesis primers [the sense primer 5’-AAG AAA AAG TGG *A* AT TTA GAA ATC TC-3’ and anti-sense primer 5’-GAG ATT TCT AAA *T* TC CAC TTT TTC TT-3’ (underlined and italicized letter is the intentional mutation)] were used to change the amino acid residue from 324D to 324N in the FcγRIA expression construct. The sequences of all the cloned constructs were confirmed by direct sequencing from both directions on an ABI 377 Sequencer with ABI BigDye Terminator Cycle Sequencing Kit.

### Generation of Stable Cell Lines Expressing FcγRIA (CD64)

Pseudo-viral particles of pCDH-MSCV-MCS-IRES-copGFP expression constructs were produced with the packaging plasmids according to the vendor’s manual (System Biosciences). The murine macrophage cell line P388D1 (P388) and rat mast cell line RBL-2H3 (RBL) obtained from ATCC (Manassas, VA) were maintained in the DMEM medium supplemented with 10% fetal calf serum (FBS) and L-glutamine (2 mM). Transductions of lentiviral particles were carried out on a 60-mm cell culture dish with the cell density at ~80% confluence. Pseudo-viral particles in 5 ml culture medium were mixed with polybrene (final concentration: 4 µg/ml) before being added to the target cell dishes. The medium was removed, and fresh medium was added one day after transduction. Two days after transduction, the CD64 expression was detected by FACS analyses. The transduced cells were sorted on a FACSAria II (BD Biosciences) for equal expression of all constructs in both the P388D1 and RBL-2H3 cells.

### Phagocytosis Assay

P388 cells expressing equivalent levels of either FcγRIA-p.324D (rs1050204G) or FcγRIA-p.324N (rs1050204A) were used to determine the phagocytosis capacity of two FcγRIA alleles. An adherent phagocytosis assay with the probe consisting of biotinylated bovine erythrocytes (aka EB) and biotinylated anti-CD64 mAb 32.2 F(ab′)_2_ was used to examine phagocytosis capacity of FcγRIA alleles in P388D1 cells as previously described (6, 31, 32). EB were saturated with streptavidin to form erythrocyte-biotin-avidin (aka EBA). After a wash step, the EBA were coated with biotinylated anti-CD64 mAb 32.2 F(ab’)_2_ (aka EBA-32.2) and the levels of mAb 32.2 binding to EBA were verified by flow cytometry. P388D1 cells adhered to round glass coverslips were incubated with EBA-32.3 in medium for 1 hour at 37 °C. Non-internalized bovine erythrocytes were lysed by brief immersion of the coverslip in distilled H_2_O followed by immersion in buffer. Three negative controls were included (1): the parental P388D1 cells with EBA-32.2 (2); the P388 cells expressing FcγRIA-p.324D with EBA, and (3) the P388 cells expressing FcγRIA-p.324N with EBA. Phagocytosis was quantitated using light microscopy and presented as the phagocytic index (number of bovine erythrocytes internalized per 100 P388D1 cells). At least 200 cells per slide were counted in duplicate without knowledge of the cell types.

### Degranulation Assays

RBL cells (10^5^ cells/well) expressing either FcγRIA-p.324D (rs1050204G) or FcγRIA-p.324N (rs1050204A) allele were cultured overnight in 24-well culture plates (Corning). The culture media were removed from plates and the cells were incubated with DMEM medium containing either anti human CD64 mAb F(ab’)_2_ (clone 32.2, final concentration of 5 µg/ml) or mouse IgG (mIgG) F(ab’)_2_ for 45 min at 4°C. The cells were washed with Tyrode’s buffer (130 mM NaCl, 5 mM KCl, 1.4 mM CaCl_2_, 1 mM MgCl_2_, 5.6 mM glucose, 10 mM HEPES, and 0.1% BSA, pH 7.4) and then stimulated with goat anti mouse F(ab’)_2_ (Jackson ImmunoResearch, West Grove, PA) at final concentration of 20 μg/ml in the Tyrode’s buffer. The supernatants were collected at 0, 15, 30, 45, and 60 min for measurement of β-hexosaminidase activity. Cells from control wells were lyzed with the same volume of 0.1% Triton X-100 in Tyrode’s buffer for evaluation of the total β-hexosaminidase activity for each RBL stable cell lines. Supernatants and cell lysates were incubated with substrate (1.3 mg/ml p-nitrophenyl-N-acetyl β-D-glucosamine) (Sigma, St. Louis, MO) in 0.1 mM sodium citrate (pH 4.5) for 1 hour at 37°C. The reaction was stopped by 0.2 mM sodium carbonate buffer (pH 10.0) and the enzyme reactivity was evaluated by measuring optical density at 405 nm. The percentage of specific β-hexosaminidase activity released was calculated as follows: percentage release (%) = 100 × average supernatant activity from 4 wells/average cell lysate activity from 4 control wells.

### Cytokine Analysis

P388 cells were stimulated in 24-well tissue culture plates (Corning) with surface-bound anit-CD64 mAb 32.2 F(ab’)_2_ as previously described (33). Wells were coated with either anti-CD64 mAb 32.2 F(ab’)_2_ (20 μg/ml) or control mIgG F(ab’)_2_ (20 μg/ml) overnight at room temperature. The CD64-expressing cells were added to coated wells and the culture media were collected after 24 hours. The levels of murine Il-1β, IL-6, or TNFα in culture media were quantified by BD™ Cytometric Bead Array (CBA) kits (cat# 558279 for murine Il-1β, cat# 558301for IL-6, and cat# 558299 for TNFα respectively, BD Biosciences).

### TaqMan Genotyping Assays of *FCGRIA* Variants

High-throughput TaqMan genotyping assays of *FCGR1A* variants were used to determine genotypes of human subjects. Two *FCGR1A* gene-specific DNA fragments (the 1,009-bps fragment containing the SNV rs1848781 and the 2,131-bps fragment containing the indel variant rs587598788 and the SNV rs1050204) were amplified by PCR using genomic DNA and primers listed on **Table 1**. Standard TaqMan reactions were subsequently performed with 1 µl of the respective *FCGR1A* gene-specific PCR products, the primers, and fluorescence-labeled (FAM or Vic) labeled probes (**Table 1**). The *FCGR1A* genotypes were determined using Applied Biosystems 7500 Software. The TaqMan assay genotypes were compared to those determined by Sanger sequencing methodology. A perfect (100%) concordance of genotypes between TaqMan assay and direct sequencing analysis was achieved in all 102 human subjects, confirming the specificity and accuracy of *FCGR1A* TaqMan genotyping assays.

**Table 1:** Primers and probes of TaqMan allele discrimination assays for *FCGR1A* variants.

Variant ID (Gene region)	Gene-specific primers (5’ to 3’)	TaqMan Primers and Probes (5’ to 3’)
**rs1848781 (c.-131C>G)**	F: TCTTTAGCTCTCTTTTTTTAGCTCTCA	AAA GCATGTTTCAAGAATTTGAGATG
(Promoter)	R: CTTTTCATAAAATAAGCTCTAATAAACA	CAAATTAGAAAAGAGGAAGGAAATTGC
	(Gene-sepcific PCR production length: 1,009 bps)	FAM- TTCCCAGAA AAG *C* AA CAT
		Vic- AGA AAA G *G* A ACA TGA TG
**rs587598788 (c.845-23_845-**	F: GTGCTTGGTGAGTGAGAATGAC	CCCTAGCTCCCAGCTCTTCA
**17delTCTTTG)**	R: GTTCAGTTTTTACCTCAGCTATGT	TGCAGTAGATCAAGGCCACTACA
(Intron 5)	(Gene-sepcific PCR production length: 2,131 bps)	FAM- AGTATCTCTTCTC *TTTGTC* T
		Vic- TAGTATCTCTTCTCTTTTTCTG
**rs1050204 (c.970G>A)**	F: GTGCTTGGTGAGTGAGAATGAC	TGG GTG ACA ATA CGT AAA GAA CTG A
(Exon 6)	R: GTTCAGTTTTTACCTCAGCTATGT	TCA TGA CCA GAA TCC AAA GAG ATT T
	(Gene-sepcific PCR production length: 2,131 bps)	FAM- AAAGAAAAAGTGG *A* ATT
		Vic- AGAAAAAGTGG *G* ATTTA

Italic and underlined nucleotides are variant sites in respective FCGR1A gene.

### Statistical Analyses

To determine associations between individual *FCGR1A* variants and sarcoidosis susceptibility, additive logistic regressions were performed either with or without race, age, and sex as covariates for each *FCGR1A* variant. Odds ratios with 95% confidence intervals were computed using the profile likelihood method of The R Project for Statistical Computing (version 4.1.2, https://www.R-project.org/). To account for the multiple testing corrections, the FDR-corrected P-values were generated by using False Discovery Rate (FDR) adjustment. For haplotype analyses, additive haplotype models were fit using logistic method, either without or with covariates. Odds ratios, p-values, and 95% confidence intervals were reported. All calculations performed using the haplo.stats R package version 1.7.9. (haplo.stats: Statistical Analysis of Haplotypes with Traits and Covariates when Linkage Phase is Ambiguous. https://CRAN.R-project.org/package=haplo.stats).

The rst_pfts (restriction on pulmonary function tests) positive (rst_pfts+) patients had worse or poorer lung functions than rst_pfts negative (rst_pfts-) patients. To determine associations between individual *FCGR1A* variant and sarcoidosis lung functions, we divided sarcoidosis patients into two groups based on rst_pfts status. Additive logistic regressions were performed for each *FCGR1A* variant with rst_pfts status (positive or negative) as response variables. Odds ratios with 95% confidence intervals were computed using the profile likelihood method of The R Project for Statistical Computing.

Unpaired t-tests (Mann-Whitney test) were used to analyze CD64 expression levels on monocytes and neutrophils between sarcoidosis patients and healthy controls and among healthy donors stratified with *FCGR1A* variant genotypes. The Student’s t-test was used to analyze the data for promoter reporter, cytokine production, and degranulation assays. A *P*-value less than 0.05 was considered as significant.

## Results

### Identification of *FCGR1A* Variants

*FCGR1A* gene contains six exons. The exon 1 and 2 (S1 and S2) code for the CD64 signal peptide while the exon 3, 4, and 5 code for the extracellular domain 1 (EC1), 2 (EC2), and 3 (EC3), respectively. The CD64 transmembrane segment and cytoplasmic domain (TMC) are coded by the exon 6 (**Figure 1**). The full-length *FCGR1A* gene fragment (11,685 bps) containing proximal promoter and all six exons was amplified with a long-template PCR and subsequently analyzed by direct Sanger sequencing. By sequencing the *FCGR1A* promoter and exons of 102 human subjects, we did not detect any non-synonymous SNVs within *FCGR1A* exons coding for three CD64 extracellular domains responsible for the interaction with IgG ligands. On the other hand, as shown in **Figure 1**, a common SNV at the nucleotide position -131 (rs1848781 or c.-131C>G) was identified in the *FCGR1A* proximal promoter region. In addition, a sole non-synonymous SNV (rs1050204 or c.970G>A) that changes the amino acid codon position 324 (p.D324N) from aspartate (D) to asparagine (N) in the FcγRIA cytoplasmic domain was identified in the exon 6. Furthermore, we detected a novel indel variant (rs587598788 or c.845-23_845-17delTCTTTG) within intron 5 that causes six-nucleotide insertion or deletion near the splicing acceptor site of the exon 6. The genotype distributions of those three variants were consistent with the Hardy-Weinberg equilibrium in 102 healthy blood donors (*P* > 0.05).

### Association of *FCGR1A* Variants With CD64 Expressions

**Figure 2:**
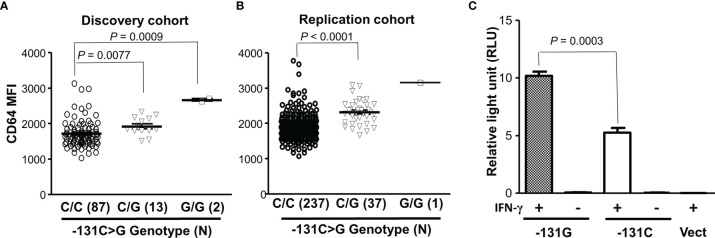
Effect of the SNV rs1848781 (c.-131C>G) on CD64 expressions. **(A)** Monocytes from SNV rs1848781 -131G/G homozygous donors expressed significantly higher levels of CD64 than those from -131C/C homozygous donors (Mann-Whitney test, *P* = 0.0009). Monocytes from -131C/G heterozygous donors also expressed significantly higher levels of CD64 than those from -131C/C homozygous donors (*P* = 0.0077). **(B)** The phenotype/genotype analysis of CD64 expressions in *FCGR1A* genotyped donors was confirmed with a replication cohort. **(C)** The promoter reporter construct containing -131G allele had significantly higher promoter activities than that with the -131C allele in the presence of IFN-γ. The -131G allele and the -131C allele had low baseline promoter activities (relative light units < 0.1) without IFN-γ stimulation, similar to the vector control (Vect). Data represent means ± SEM from four independent experiments.

To examine relationship between *FCGR1A* variant genotypes and CD64 expressions, we carried out genotype-phenotype analyses in healthy blood donors. In a discovery cohort, we found that the *FCGR1A* SNV rs1848781 (c.-131C>G) genotypes were significantly associated with CD64 expressions on resting monocytes of healthy blood donors (**Figure 2** **)**. Monocytes from C/G (-131C/G) heterozygous donors expressed significantly higher levels of CD64 than those from the C/C (-131C/C) homozygous donors (*P* = 0.0077). Monocytes from two G/G (-131G/G) homozygous donors had the highest average levels of CD64 among three genotype groups (**Figure 2**). The significant association of *FCGR1A* SNV rs1848781 (c.-131C>G) genotypes with CD64 expression levels was confirmed by a replication cohort consisting of 275 human subjects (*P* < 0.0001, **Figure 2**). We also carried out promoter reporter assays to determine whether the SNP -131C>G influences the promoter activity. As shown in **Figure 2**, the promoter reporter construct containing the SNV rs1848781G (or -131G) allele had significantly higher promoter activity than that with -131C allele in human monocytic U937 cells in the presence of IFN-γ, consistent with the observation that monocytes from the donors carrying -131G allele (-131C/G heterozygous and -131G/G homozygous donor) expressed higher levels of CD64 than those from the -131C/C homozygous donors. Taken together, data from ex vivo monocytes and *in vitro* promoter reporter assays demonstrate that the SNV rs1848781 (c.-131C>G) is a functional SNV affecting the gene expression.

**Figure 3:**
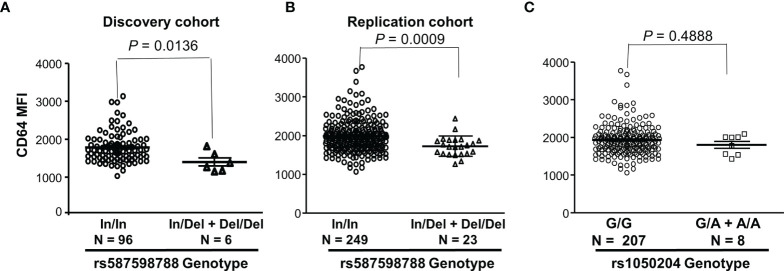
*FCGR1A* genotypes containing the rs587598788-c.845-17delTCTTTG (rs587598788Del) allele are significantly associated with low CD64 expressions. **(A)** Monocytes from rs587598788-c.845-23 (rs587598788In) homozygous donors (In/In) expressed significantly higher levels of CD64 than those from the combined rs587598788 heterozygous (In/Del) and rs587598788Del homozygous (Del/Del) donors (Mann-Whitney test *P* = 0.0136). **(B)** CD64 expression data from a replication cohort confirmed that rs587598788-In/In homozygous donors expressed significantly higher levels of CD64 than those from the combined rs587598788-In/Del heterozygous and Del/Del homozygous donors. **(C)** CD64 expression levels on monocytes were not significantly different between the SNV rs1050204-G/G homozygous donors (G/G, N = 207) and the combined rs1050204-G/A heterozygous and rs1050204-A/A homozygous donors (Mann-Whitney test *P* = 0.488).

**Figure 4:**
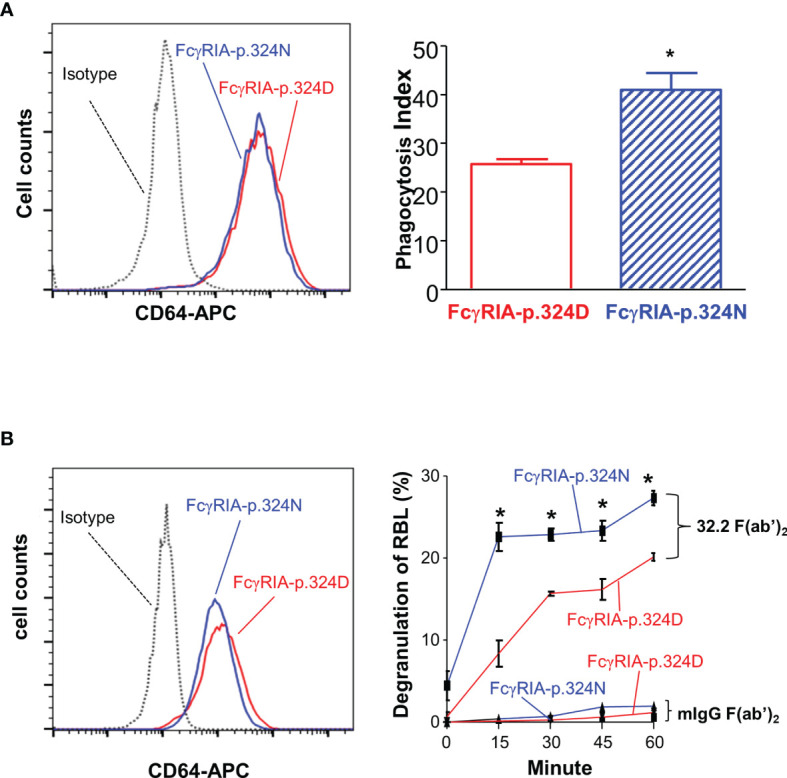
*FCGR1A* SNV rs1050204 (c.970G>A or p.D324N) alleles affect CD64-mediated phagocytosis and degranulation. **(A)** Effect of the SNV rs1050204 (FcγRIA-p.D324N) on FcγRIA-mediated phagocytosis. P388D1 cell lines expressed equivalent levels of FcγRIA-p.324D (rs1050204G) and FcγRIA-p.324N (rs1050204A) (left panel). FcγRIA-p.324N mediated significantly higher levels of phagocytosis (*Student’s t-test *P* = 0.0056, n = 4). **(B)** Effect of the SNV rs1050204 (p.D324N) on FcγRIA-mediated degranulation. Stably transfected and RBL cell lines express FcγRIA-p.324D (rs1050204G) and FcγRIA-p.324N (rs1050204A) alleles at comparable levels. FcγRIA-mediated degranulation induced by anti-CD64 mAb 32,2 F(ab’)_2_ crosslinking was significantly higher in the RBL cells with FcγRIA-p.324N allele than those with FcγRIA-p.324D (*Student’s t-test, *P* < 0.01, n = 4). No degranulation was induced by the irrelevant mIgG F(ab’)_2_ in the stable RBL cells expressing either p.324D and p.324N alleles.

We further analyzed effects of *FCGR1A* rs587598788 and rs1050204 variants on CD64 expressions. As shown in **Figure 3**, resting monocytes from the rs587598788-c.845-23 (rs587598788In) homozygous donors (In/In) expressed significantly higher levels of CD64 than the combined group of rs587598788 heterozygous (In/Del) and rs587598788-c.845-17delTCTTTG (rs587598788Del) homozygous donors (Del/Del) (*P* = 0.0136). The significant association between CD64 expression levels and rs587598788 genotypes was confirmed in a replication cohort (*P* = 0.0009, **Figure 3**). On the other hand, the SNV rs1050204 genotypes were not associated with CD64 expression (*P* = 0.4888, **Figure 3**), which was consistent with the equivalent or similar expression of CD64 in the cell lines expressing rs1050204G (FcγRIA-p.324D) and rs1050204A (FcγRIA-p.324N) alleles (**Figures 4A, B**).

### The *FCGR1A* SNV rs1050204 Alleles Affect FcγRIA-Mediated Immune Functions

**Figure 5:**
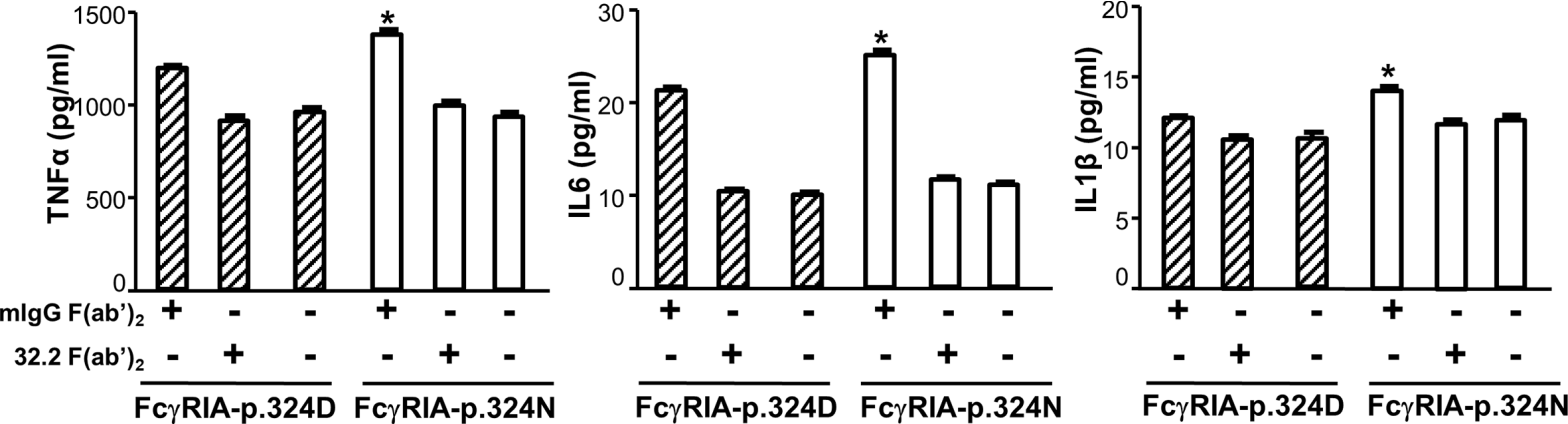
*FCGR1A* SNV rs1050204 (FcγRIA-p.D324N) alleles affect CD64-mediated cytokine productions. FcγRIA-mediated IL-6, IL-1β, and TNFα productions were significantly higher in P388 cells with the FcγRIA-p.324N (rs1050204A) allele than those with the FcγRIA-p.324D allele (rs1050204G) (*Student’s t-test *P* < 0.01, n = 4). IL-6, IL-1β, and TNFα productions were not different between the FcγRIA-p.324D and the FcγRIA-p.324N alleles in mIgG F(ab’)_2_-stimulated cells or untreated cells (*P* > 0.05).

Previous studies demonstrated that FcγRIA cytoplasmic domain influences receptor functions (6, 32, 34). The *FCGR1A* SNV rs1050204 (c.970G>A or p.D324N) leads to non-conservative residue change from p.324D to p.324N in the FcγRIA cytoplasmic domain that participates in signal transduction. Consequently, we investigated whether the *FCGR1A* SNV rs1050204 (FcγRIA-p.D324N) alleles affect FcγRIA-mediated functions using P388D1 cell lines expressing equivalent levels of FcγRIA-p.324D (rs1050204G) and FcγRIA-p.324N (rs1050204A). Phagocytosis assay was carried out as described in “Materials and Methods” and all negative controls showed no phagocytosis of bovine erythrocytes. FcγRIA-p.324N mediated significantly higher levels of phagocytosis (phagocytosis index = 41.00 ± 3.46) than the FcγRIA-p.324D (phagocytosis index = 25.75 ± 1.03) (*P* = 0.0056) (**Figure 4**). In addition, the FcγRIA-p.324N allele mediated significantly more degranulation than the FcγRIA-p.324D allele (**Figure 4**) and significantly more pro-inflammatory cytokine (IL-6, IL-1β, and TNFα) productions than did the FcγRIA-p.324D allele (**Figure 5**). Our data demonstrate that the *FCGR1A* SNV rs1050204 (FcγRIA-p.D324N) within CD64 cytoplasmic domain significantly affect receptor-mediated functions, similar to the effect of the SNV within FcαRI cytoplasmic domain (33).

### Association of *FCGR1A* Variant Haplotypes With Sarcoidosis Susceptibility

We subsequently carried out a genetic study to examine whether *FCGR1A* variants are associated with sarcoidosis susceptibility using the ACCESS cohort subjects. As previously described, the major goal of ACCESS was to address the hypotheses that sarcoidosis occurs in genetically susceptible individuals through alteration in immune response after exposure to an environmental, occupational, or infectious agents (35, 36). Specific phenotypes of sarcoidosis were determined with an instrument developed by the ACCESS group (37). The clinical characteristics of the study patients have been described previously (38). As shown in **Table 2**, *FCGR1A* SNV rs1020204 genotypes were associated with sarcoidosis susceptibility (*P* = 0.042, *P_FDR_* = 0.111, OR 1.222, 95% CI 1.007 – 1.546) while rs587598788 genotypes tended to associate with sarcoidosis (*P* = 0.073, *P_FDR_* = 0.111, OR 1.431, 95% CI 0.962 – 2.128). On the other hand, SNV rs1848781 genotypes were not associated with sarcoidosis risk (*P* = 0.747) (**Table 2**).

**Table 2:** Association of *FCGRIA* varaints with sarcoidosis susceptibility.

Variant	Risk allele	Genotype Frequency (%)			Unadjusted*			Adjusted for age and sex*		
					*P*	*P_FDR_*	OR (95% CI)	*P*	*P_FDR_*	OR (95% CI)
**rs1848781**	**C**	**C/C**	**C/G**	**G/G**						
Case (N = 670)	1056 (78.8%)	439 (65.5%)	178 (26.6%)	53 (7.9%)	0.769	0.769	1.026 (0.867-1.214)	0.747	0.747	1.030 (0.857-1.239)
Control (N = 669)	1048 (78.3%)	434 (64.9%)	180 (26.9%)	55 (8.2%)						
**rs587598788**	**In**	**In/In**	**In/Del**	**Del/Del**						
Case (N = 670)	1301 (97.1%)	633 (47.2%)	35 (2.6%)	2 (0.1%)	0.077	0.115	1.418 (0.959-2.096)	0.074	0.111	1.431 (0.962-2.128)
Control (N = 669)	1281 (95.7%)	618 (46.2%)	45 (3.4%)	6 (0.4%)						
**rs1050204**	**G**	**G/G**	**A/G**	**A/A**						
Case (N = 670)	1122 (83.7%)	474 (70.7%)	174 (26.0%)	22 (3.3%)	0.044	0.115	1.217 (1.005-1.475)	0.042	0.111	1.222 (1.007 -1.546)
Control (N = 669)	1079 (80.6%)	446 (66.7%)	187 (28.0%)	36 (5.4%)						

*Additive model was used to test mode of inheritance.

*FCGR1A* variants in linkage disequilibrium could form different haplotypes to impact gene functions. Subsequently, we carried out haplotype analysis to examine whether *FCGR1A* variant haplotypes are associated with the sarcoidosis susceptibility. As shown in **Table 3**, we found that the haplotype C-Del-A (rs1848781C-rs587598788Del-rs1050204A) was significantly associated with the protection against sarcoidosis development (logistic regression adjusted for sex and age, *P* = 0.008, OR 0.542, 95% CI 0.346 – 0.853). The frequency of the haplotype C-Del-G (rs1848781C-rs587598788Del-rs1050204G) containing the rs1848781C-rs587598788Del also tended to be lower in sarcoidosis patients (1.95%) than that in the matched controls (2.4%). Taken together, our data suggest that *FCGR1A* haplotypes may affect the pathogenesis of sarcoidosis.

**Table 3:** Association of *FCGRIA* variant haplotypes (rs1848781-rs587598788-rs1050204) with sarcoidosis susceptibility.

Haplotypers1848781-rs587598788-rs1050204	Estimated Frequency (%)		Logistic regression		Logistic regression adjusted for sex and age	
	Case (N = 670)	Control (N = 669)	P value*	OR (95% CI)	P value*	OR (95% CI)
C-In-G	74.7%	71.6%				
G-In-A	12.7%	11.5%	0.261	0.877 (0.698-1.102)	0.267	0.876 (0.693-1.107)
G-In-G	8.9%	9.0%	0.827	0.973 (0.758-1.247)	0.826	0.969 (0.735-1.278)
C-Del-A	2.3%	4.2%	0.008	0.542 (0.346-0.849)	0.008	0.543 (0.346-0.853)
C-Del-G	1.9%	2.4%	0.306	0.765 (0.458-1.277)	0.313	0.762 (0.450-1.292)

*The p-values for the estimated haplotype were generated using the most common haplotyoe rs1848781C-rs587598788In-rs1050204G as the reference for the logistic regression analyses.

### Association of *FCGR1A* Variants With Poor Lung Function in Sarcoidosis Patients

Sarcoidosis frequently affects lung functions (14). We analyzed whether *FCGR1A* variants are associated lung functions among sarcoidosis patients. The restriction on pulmonary function tests (rst_pfts) status was used to stratify sarcoidosis patients as described in “*Materials and Methods*”. As shown in **Table 4**, *FCGR1A* rs1848781 genotypes were significantly associated with the risk for poor lung function (genotype C/G+G/G vs C/C, *P* = 0.001, *P_FDR_* = 0.016, OR 1.695, 95% CI 1.263 – 2.262). In addition, the rs1050204 genotypes tended to associate with the risk for poor lung function as well (genotype A/G+A/A vs G/G, *P* = 0.035, *P_FDR_* = 0.263, OR 1.471, 95% CI 1.028 – 2.079). On the other hand, no significant difference in the distribution of rs587598788 genotypes was observed between rst_pfts positive and rst_pfts negative sarcoidosis patients, possibly due to the low frequency of the rs587598788Del allele in populations. Our data suggest *FCGR1A* variant genotypes are risk factors for poor lung function in sarcoidosis patients, pointing to a role of *FCGR1A* in lung inflammation.

**Table 4:** Association of *FCGRIA* variants with poor lung function among sarcoidosis patients.

Variant	Risk allele	Genotype Frequency (%)			P*	P_FDR*_	OR (95% CI)
**rs1848781**	G	C/C	C/G	G/G			
rst_pfts+ (N = 113)	70 (31.0%)	58 (51.3%)	40 (35.4%)	15 (13.3%)	0.001	0.016	1.695 (1.263-2.262)
rst_pfts- (N = 557)	214 (19.2%)	381 (68.4%)	138 (24.8%)	38 (6.8%)			
**rs587598788**	In	In/In	In/Del	Del/Del			
rst_pfts+ (N = 113)	222 (98.2%)	110 (97.3%)	2 (1.8%)	1 (0.9%)	0.384	0.384	1.800 (0.633-5.118)
rst_pfts- (N = 557)	1079 (96.9%)	523 (93.9%)	33 (5.9%)	1 (0.2%)			
**rs1050204**	A	G/G	A/G	A/A			
rst_pfts+ (N = 113)	48 (21.2%)	71 (62.8%)	36 (31.9%)	6 (5.3%)	0.035	0.263	1.471 (1.028-2.079)
rst_pfts- (N = 557)	170 (15.3%)	403 (72.4%)	138 (24.8%)	16 (2.9%)			

*Additive model was used to test mode of inheritance. rst_pfts: restriction on pulmonary function tests, rst_pfts positivity indicates a poor lung function.

### Increased CD64 Expressions on Monocytes and Neutrophils in Sarcoidosis Patients

**Figure 6:**
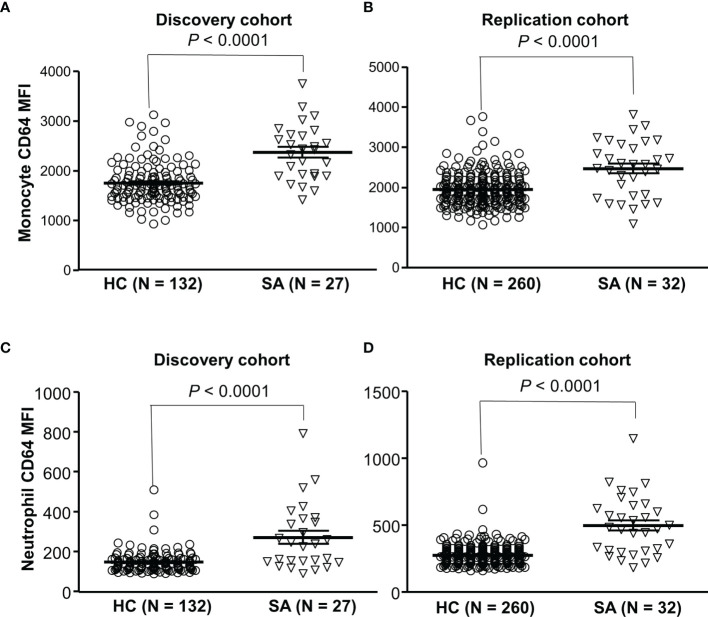
Increased CD64 expressions on monocytes and neutrophils in sarcoidosis patients. Monocytes from sarcoidosis patients (SA) expressed significantly higher levels of CD64 than those from healthy controls (HC) in both discovery cohort **(A)** and replication cohort **(B)** (Mann-Whitney test *P* < 0.0001). Neutrophils from sarcoidosis patients (SA) expressed significantly higher levels of CD64 than those from healthy controls (HC) in both discovery cohort **(C)** and replication cohort (Mann-Whitney test *P* < 0.0001) **(D)**.

To investigate a role of FcγRIA in sarcoidosis, we examined expression levels of CD64 in sarcoidosis patients and healthy blood donors. We found that CD64 expressions on monocytes (**Figures 6A, B**) and neutrophils (**Figures 6C, D**) were significantly increased in sarcoidosis patients (SA) as compared to healthy controls (HC) (*P* < 0.001). Our data indicate that the upregulation of CD64 could be a biomarker for sarcoidosis.

## Discussion

In the current study, we failed to detect any non-synonymous SNVs within all *FCGR1A* exons (the exon 3, 4, and 5) coding for CD64 extracellular domains that are responsible for binding to IgG ligands. Our data indicate that the selection pressure to maintain the high affinity interaction between CD64 and IgGs may prevent the fixation of any detrimental mutations within FcγRIA extracellular domains during human evolution. On the other hand, sequence analyses of *FCGR1A* promoter and exons revealed two common *FCGR1A* SNVs (rs1848781 and rs1050204) and an indel variant (rs587598788). *FCGR1A* variants significantly affected either FcγRIA expression levels or receptor-mediated functions. Genetic analyses demonstrated that the *FCGR1A* SNV rs1050204 genotypes were associated with sarcoidosis susceptibility while the C-Del-A (rs1848781C-rs587598788Del-rs1050204A) haplotype was significantly associated with the protection against sarcoidosis. Our data suggest that functional *FCGR1A* variants may play important role in the pathogenesis of sarcoidosis.

In the genotype-phenotype analyses, we found that the genotypes containing *FCGR1A* SNV rs1848781G (or -131G) allele were significantly associated with high levels of CD64 expression on resting monocytes. Additionally, *FCGR1A* promoter activity of the SNV rs1848781G allele was significantly higher than that of the rs1848781C allele in promoter reporter assays, consistent with the concept that *FCGR1A* promoter with rs1848781G allele could drive higher CD64 expression than the rs1848781C allele in monocytes. We conclude that the SNV rs1848781C>G is a functional *FCGR1A* polymorphism. Concomitantly, genotypes containing the high activity rs1848781G (-131G) allele were significantly associated with restriction on pulmonary function tests in sarcoidosis patients, suggesting that rs1848781G allele is a risk factor for poor lung functions.

*FCGR1A* SNV rs1050204 (p.D324N) genotypes were significantly associated with sarcoidosis susceptibility and poor lung functions. After False Discovery Rate (FDR) adjustment for multiple tests, the associations of rs1050204 genotypes with sarcoidosis phenotypes were not statistically significant. To predict whether the SNV rs1050204 (p.D324N) is a detrimental mutation for human genetic diseases; we carried out the in silico analysis using PredictSNP1 tool (39). Analysis results from PredictSNP, MAPP, PhD-SNP, PolyPhen-1, PolyPhen-2, SIFT, and SNAP predict that the SNV rs1050204 (p.D324N) is a neutral variant with the prediction accuracy between 67% and 98%. FcγRIA-p.324N seems to be a gain-of-function allele in comparison to the FcγRIA-p.324D while non-synonymous SNV is absent in FcγRIA extracellular domains. We speculate that the selection pressure for strong immune responses may prevent the fixation of any detrimental mutations within human *FCGR1A* coding region during human evolution. Nevertheless, our functional assays revealed that the p.324N allele mediated significantly more productions of pro-inflammatory cytokines (IL6, IL1β, and TNFα) and more FcγRIA-mediated degranulation than the p.324D allele, suggesting that the pro-inflammatory p.324N allele may have a role in poor lung functions. The SNV rs1050204 (p.D324N) in the CD64 cytoplasmic domain leads to non-conservative amino acid change (p.324D → p.324N). The SNV FcγRIA-p.D324N is located within the interactions region between FcγRIA cytoplasmic domain and the cytoskeletal molecules periplakin (40, 41) or protein 4.1G (34, 42), which may explain the functional differences between two allele. Therefore, the non-detrimental SNV rs1050204 (p.D324N) could only serve as a disease modifier for sarcoidosis. The precise molecular mechanisms underlying the effect of the SNV FcγRIA-p.D324N on FcγRIA-mediated functions required further investigation.

*FCGR1A* gene produces the high-affinity IgG Fc receptor FcγRIA (or CD64) capable of binding monomeric IgG (12, 13). CD64 plays a critical role in inflammation. CD64 is sufficient to trigger autoimmune arthritis, thrombocytopenia, immune complex-induced airway inflammation, and active and passive systemic anaphylaxis *in vivo* (43). Mouse models clearly demonstrated that CD64 significantly influences inflammatory responses (44, 45). Most importantly, activation of CD64 induces the differentiation of monocytes into specialized immature dendritic cells with the capacity to expand autoreactive T cell responses (46), which contribute to inflammatory disorders (27, 47, 48). In sarcoidosis patients, elevated circulating IgG and immune complexes are strongly associated with disease activities, especially in patients with pulmonary sarcoidosis (49–52). A distinctive feature of sarcoidosis is that CD4^+^ T cells interact with antigen-presenting cells to initiate the formation and maintenance of granulomas (53). Sarcoidosis is frequently associated with humoral abnormalities such as hypergammaglobulinemia (49, 52), autoantibody production (54), and circulating immune complexes (51), suggesting that abnormal antigen presentations and antibody productions are involved in the pathogenesis of sarcoidosis. CD64 is constitutively expressed on antigen-presenting cells such as monocytes, macrophages, and dendritic cells and facilitates MHC-class II-mediated antigen presentation (55). CD64 mediates the most effective antigen presentation *in vitro* (4, 56) and *in vivo* (57). CD64 samples constant sources of extracellular antigens through internalizing IgG immune complexes (58) and is considered as an effective adjuvant target for vaccination (4, 56, 57). Phenotypically, we found that CD64 expressions on monocytes from sarcoidosis patients were significantly increased compared to those from healthy controls, indicating that elevated CD64 expressions and enhanced CD64 functions may facilitate the development of sarcoidosis.

Genotypes of the rs587598788 indel variant were also significantly associated with levels of CD64 expression, indicating that the rs587598788 allelic variants may affect splicing or maturation of full-length *FCGR1A* mRNA. A limitation of the current study is that the effect of rs587598788 variant alleles on *FCGR1A* mRNA level was not examined. Further study is required to determine whether the rs587598788 alleles influence *FCGR1A* mRNA splicing and stability, which will provide a molecular mechanism of the association between rs587598788 genotypes and the levels of CD64 protein expression.

The genotypes containing rs587598788Del allele were significantly associated with low CD64 expression on resting monocytes from healthy blood donors, which may explain the association of the haplotype C-Del-A (rs1848781C- rs587598788Del-rs1050204A) haplotype with the protection against sarcoidosis. The haplotype C-Del-G (rs1848781C- rs587598788Del-rs1050204G) haplotype also tended to increase in sarcoidosis patients but did not reach a significant level (**Table 3**), likely due to the low allele frequency in human populations. Our data strongly suggest that the decreased CD64 expressions have a protective role against the development of sarcoidosis. Therefore, blockade of CD64 functions or prevention of CD64 upregulation may be a useful therapeutic strategy in the treatment of sarcoidosis. Future studies are required to pinpoint the precise mechanisms of CD64 in the pathogenesis of sarcoidosis.

## Conclusion

*FCGR1A* genetic variants affect CD64 expression and functions, which could play important roles in the development and manifestation of sarcoidosis. *FCGR1A* variants may serve as a biomarker for sarcoidosis susceptibility and severity. Targeting CD64 may be an effective option for the treatment of sarcoidosis.

## Data Availability Statement

The original contributions presented in the study are included in the article/supplementary material, further inquiries can be directed to the corresponding author.

## Ethics Statement

The studies involving human participants were reviewed and approved by The Institutional Review Board for Human Use at the University of Minnesota (IRB Protocol #1301M26461). The patients/participants provided their written informed consent to participate in this study.

## Author Contributions

All authors contributed to the study conception and design. Conceptualization, resources, and funding acquisition: JW and MB. Methodology, investigation, data acquisition and analyses: JW, YL, AR, and MB. Manuscript preparation: JW and MB. All authors contributed to the article and approved the submitted version.

## Funding

This study was supported by National Institute of Health grant R21 AI125729 and R21 AI149395. The funders had no role in study design data collection and analysis, decision to publish, or preparation of the manuscript.

## Conflict of Interest

The authors declare that the research was conducted in the absence of any commercial or financial relationships that could be construed as a potential conflict of interest.

## Publisher’s Note

All claims expressed in this article are solely those of the authors and do not necessarily represent those of their affiliated organizations, or those of the publisher, the editors and the reviewers. Any product that may be evaluated in this article, or claim that may be made by its manufacturer, is not guaranteed or endorsed by the publisher.

## Supplementary Material

Supplementary Figure 1 Structure of FcγRIA and location of non-synonymous *FCGR1A* SNV rs1050204 (FcγRIA-p.D324N). **(A)** FcγRIA (CD64) contains three extracellular domains (EC1, EC2, and EC3), a transmembrane segment, and a cytoplasmic domain. The non-synonymous SNV (FcγRIA-p.D324N) is located at the membrane proximal region of cytoplasmic domain. **(B)** Illustration of the exact location of the SNV FcγRIA-p.D324N on the peptide of FcγRIA transmembrane segment and cytoplasmic domain.
