# Supplementary figures and images for: Novel Human FCGR1A Variants Affect CD64 Functions and Are Risk Factors for Sarcoidosis
Source: Front Immunol. 2022 Mar 17;13:841099. doi: 10.3389/fimmu.2022.841099 (PMC8968912; doi:10.3389/fimmu.2022.841099)

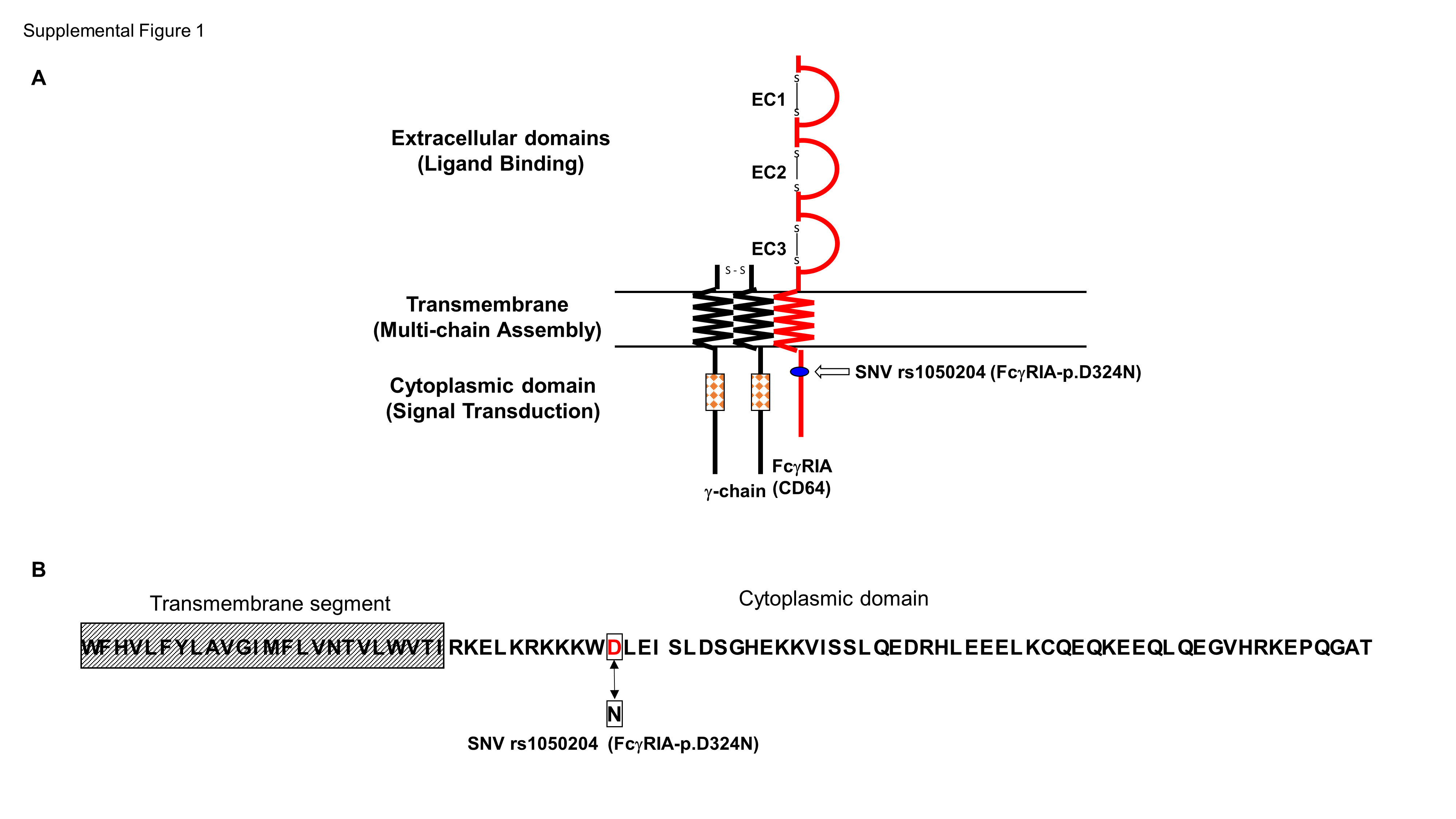

Supplement: Supplementary Figure 1 — Structure of FcγRIA and location of non-synonymous FCGR1A SNV rs1050204 (FcγRIA-p.D324N). (A) FcγRIA (CD64) contains three extracellular domains (EC1, EC2, and EC3), a transmembrane segment, and a cytoplasmic domain. The non-synonymous SNV (FcγRIA-p.D324N) is located at the membrane proximal region of cytoplasmic domain. (B) Illustration of the exact location of the SNV FcγRIA-p.D324N on the peptide of FcγRIA transmembrane segment and cytoplasmic domain. [file Image_1.tif]
